# ARTP Mutagenesis of *Schizochytrium* sp. PKU#Mn4 and Clethodim-Based Mutant Screening for Enhanced Docosahexaenoic Acid Accumulation

**DOI:** 10.3390/md19100564

**Published:** 2021-10-07

**Authors:** Lu Liu, Mohan Bai, Sai Zhang, Jiantao Li, Xianhua Liu, Biswarup Sen, Guangyi Wang

**Affiliations:** 1 Center for Marine Environmental Ecology, School of Environmental Science and Engineering, Tianjin University, Tianjin 300072, China; 1018214019@tju.edu.cn (L.L.); bmh@zju.edu.cn (M.B.); zhangsai@tju.edu.cn (S.Z.); tao076077_@tju.edu.cn (J.L.); lxh@tju.edu.cn (X.L.); 2 Polar Research Institute of China, Shanghai 200136, China; 3 Key Laboratory of Systems Bioengineering (Ministry of Education), Tianjin University, Tianjin 300072, China

**Keywords:** thraustochytrids, polyunsaturated fatty acids, mutagenesis, atmospheric and room-temperature plasma, acetyl-CoA carboxylase, clethodim

## Abstract

*Schizochytrium* species are one of the best oleaginous thraustochytrids for high-yield production of docosahexaenoic acid (DHA, 22:6). However, the DHA yields from most wild-type (WT) strains of *Schizochytrium* are unsatisfactory for large-scale production. In this study, we applied the atmospheric and room-temperature plasma (ARTP) tool to obtain the mutant library of a previously isolated strain of *Schizochytrium* (i.e., PKU#Mn4). Two rounds of ARTP mutagenesis coupled with the acetyl-CoA carboxylase (ACCase) inhibitor (clethodim)-based screening yielded the mutant A78 that not only displayed better growth, glucose uptake and ACCase activity, but also increased (54.1%) DHA content than that of the WT strain. Subsequent optimization of medium components and supplementation improved the DHA content by 75.5 and 37.2%, respectively, compared with that of mutant A78 cultivated in the unoptimized medium. Interestingly, the ACCase activity of mutant A78 in a medium supplemented with biotin, citric acid or sodium citrate was significantly greater than that in a medium without supplementation. This study provides an effective bioengineering approach for improving the DHA accumulation in oleaginous microbes.

## 1. Introduction

As one of the most important polyunsaturated fatty acids (PUFA), docosahexaenoic acid (DHA, 22:6) not only benefits the development of the fetus’s nerves and retina but also prevents several human diseases such as cardiovascular disease, hypertension and thrombi [1]. Given the multiple benefits to human health, DHA has been an important functional food component with high demand on the global market. However, due to the lack of a complete PUFA biosynthesis pathway, humans can only obtain DHA from dietary supplements. As the current major source of DHA, deep-sea fishes obtain PUFA from marine microorganisms, particularly marine microalgae and protists [2,3,4]. Unfortunately, the supply of fish oil is limited by food security, environmental and toxicity concerns and, therefore, further research is needed to develop efficient and sustainable alternative sources of DHA [5]. 

Thraustochytrids, a group of unicellular heterotrophic protists with ubiquitous presence in marine environments [6,7], are widely used in the production of DHA [8]. However, the low production yield of most thraustochytrid strains remains to be a key limiting factor for large-scale DHA production. To date, the efforts on improving the production yield of thraustochytrid strains have mostly focused on the optimization of media components, such as carbon and nitrogen sources [9,10,11,12,13] and salinity [11]. While several other efforts were focused on the manipulation of fermentation parameters, e.g., pH [14], aeration rates [15], feeding strategies [12,16] and multiple-stage cultivation strategies [17]. However, the scale of DHA production solely through the optimizations of such traditional fermentation strategies has not been quite remarkable and is largely limited by the non-availability of strains/mutants with high DHA accumulation capacity. 

Strain improvements through mutagenesis, including physical and chemical methods, are one of the most commonly used approaches to obtain mutants with desirable biological and biotechnological traits [18,19,20,21,22]. For example, the atmospheric and room-temperature plasma (ARTP) mutagenesis technology can generate microbial mutation by using helium radio frequency glow discharge plasma jets [23]. Compared with the other conventional mutation methods, ARTP has several advantages, including high efficiency, consistency, nontoxicity, environment-friendly and low cost [24]. As of now, ARTP mutagenesis technology has been used for successful microbial breeding of bacteria [25], fungi [26] and microalgae [19,27]. Furthermore, ARTP mutagenesis has shown notable improvement in the production yields of several microbial products, e.g., 1,3-propanediol [28], hydrogen production [29], high acid protease [30] and lycopene [26]. Despite so, the research on the application of ARTP technology to generate thraustochytrid mutants towards achieving a high DHA yield is still in its infancy [31].

After mutagenesis, the subsequent efficient screening method is the pivotal step to obtain mutant strains with desirable features [32]. Most conventional screening methods, such as phenotypic and morphological screening, can be tedious and time-consuming [33]. Therefore, more effective screening methods are critical for developing strains of oleaginous microbes with the ability to produce high amounts of fatty acids [32]. Acetyl-CoA carboxylase (ACCase), which catalyzes the carboxylation of acetyl-CoA to malonyl-CoA, is the first committing step in the fatty acids biosynthetic pathway [34]. Upregulation of ACCase activities has been demonstrated to significantly improve the lipid production in several oleaginous microorganisms [35,36,37,38]. Furthermore, an ACCase inhibitor (sethoxydim)-based screening strategy has been successfully used to screen mutants of *Crypthecodinium cohnii* with high DHA yields [39].

Clethodim, which has been reported as a commonly used herbicide to control grass weeds growth by inhibiting ACCase [40], was found to significantly inhibit the growth of *Schizochytrium* sp. PKU#Mn4. In this study, we applied the ARTP mutagenesis technology in combination with a screening strategy that is based on clethodim—an ACCase inhibitor—to screen for high DHA yielding mutants of *Schizochytrium* sp. PKU#Mn4. Through media optimization, including supplementation of chemical modulators/activators of fatty acids biosynthesis, DHA production of the best mutant was further improved. This study provides the first report of a bioengineering approach, integrating ARTP mutagenesis and clethodim-based screening strategy, which yielded a thraustochytrid mutant with a better capacity for DHA accumulation than its parental strain.

## 2. Results

### 2.1. Optimal ARTP Exposure Time and Clethodim Concentration

**Figure 1:**
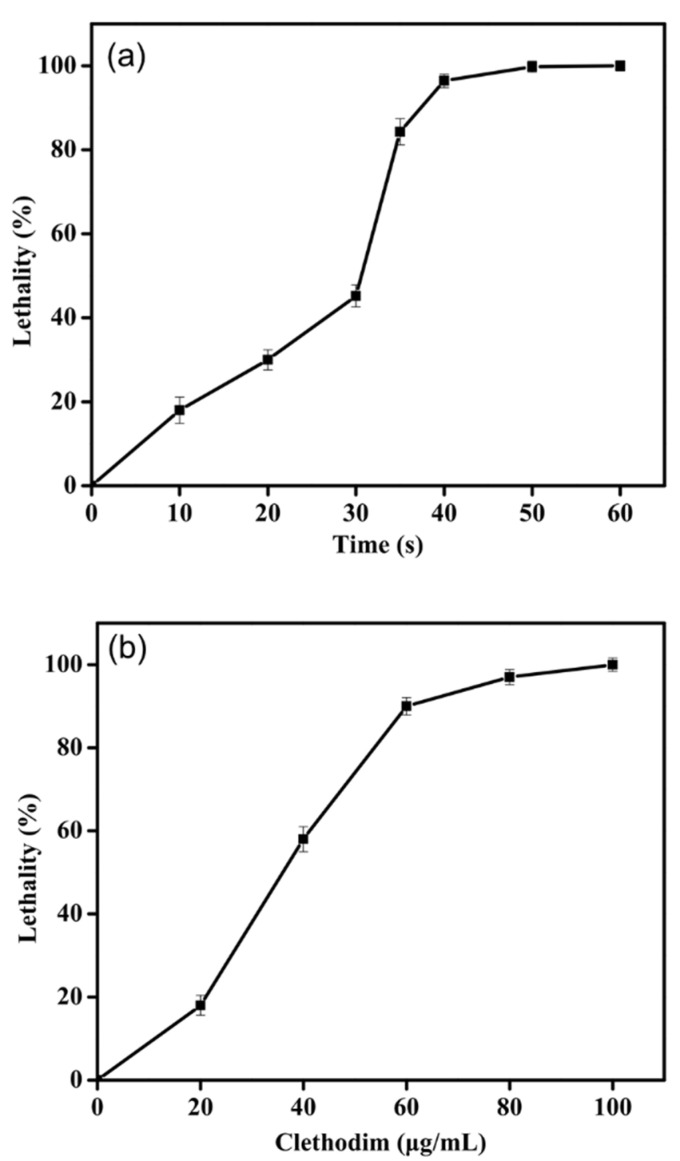
Effects of the exposure time of ARTP treatment (**a**) and clethodim levels (**b**) on the lethality of the WT strain.

The lethality (%) of ARTP treatment to the cells of strain PKU#Mn4 increased with the ARTP exposure time in a sigmoid pattern (Figure 1a). The lethality increased sharply between 30 s and 40 s and thereafter leveled off. The treatment duration of 40 s with the lethality of 96.4% was, therefore, chosen as the optimal duration time of ARTP treatment for strain PKU#Mn4. 

Clethodim addition into the culture medium resulted in the growth inhibition of strain PKU#Mn4 (Figure 1b). The number of surviving colonies decreased significantly as clethodim concentration increased. The lethality (%) of clethodim exhibited a linear increase between 0 µg/mL and 60 µg/mL and thereafter leveled off. The clethodim concentration of 80 µg/mL, which yielded 94% lethality, was used to screen the mutants with high ACCase activity. In the second round of screening, clethodim at a concentration of 95 µg/mL was used to screen the resulting mutants.

### 2.2. Mutant Screening

The first round of mutagenesis and subsequent clethodim-based screening yielded 13 mutants with better DHA content. Among these, four mutants, namely B13, B14, B15 and B22, were further selected because of their relatively higher DHA content compared with that of the WT strain (Figure S1). From the second round of ARTP mutagenesis of these four mutants followed by clethodim-based screening, 17 mutant strains with improved DHA content were obtained. The biomass and DHA contents of these 17 mutant strains were significantly higher than that of the WT strain (Table 1). In addition, the percentage of DHA in TFA was improved in all these mutants compared with that of the WT strain. Particularly, the mutants A99, A29, A74, A89, A78 and A22 were able to accumulate DHA by more than 1.5-fold and palmitic acid by more than 1.4-fold (Table S1). However, the genetic stability test indicated that only mutant A78 was stable, which showed a 54.1% increase in DHA content and was, therefore, used in further optimization experiments.

### 2.3. Dynamics of Growth, Glucose Consumption and ACCase Activity in Mutant A78

**Figure 2:**
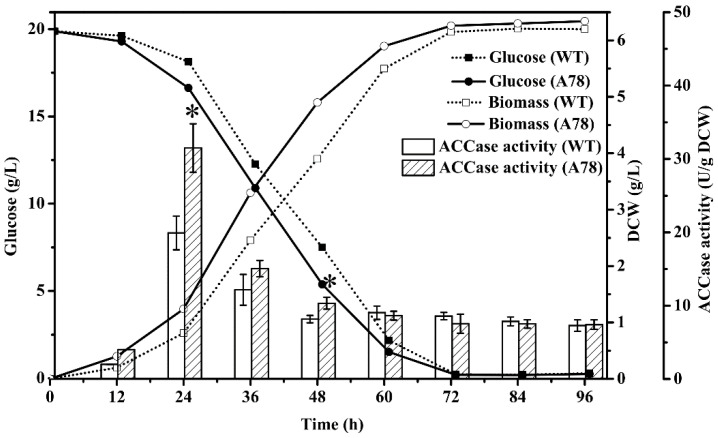
Comparison of the dynamics of biomass, glucose consumption and ACCase activity between the mutant A78 and the WT strain cultivated in the M4 medium. * *p* < 0.05.

The mutant A78 showed significantly higher growth and glucose consumption compared with the WT strain between 24 h and 60 h of cultivation in the M4 medium (Figure 2). The ACCase activity of both mutant A78 and WT strain peaked at 24 h of cultivation (Figure 2); however, the peak ACCase activity of mutant A78 was 1.6-fold that of WT strain. Moreover, the ACCase activity of mutant A78 was specifically higher than that of the WT strain between 12 and 24 h of cultivation. In addition, after 60 h of cultivation, the ACCase activity of mutant A78 remained slightly lower than or close to that of the WT strain.

### 2.4. Effects of Media Components on DHA Accumulation in Mutant A78

**Figure 3:**
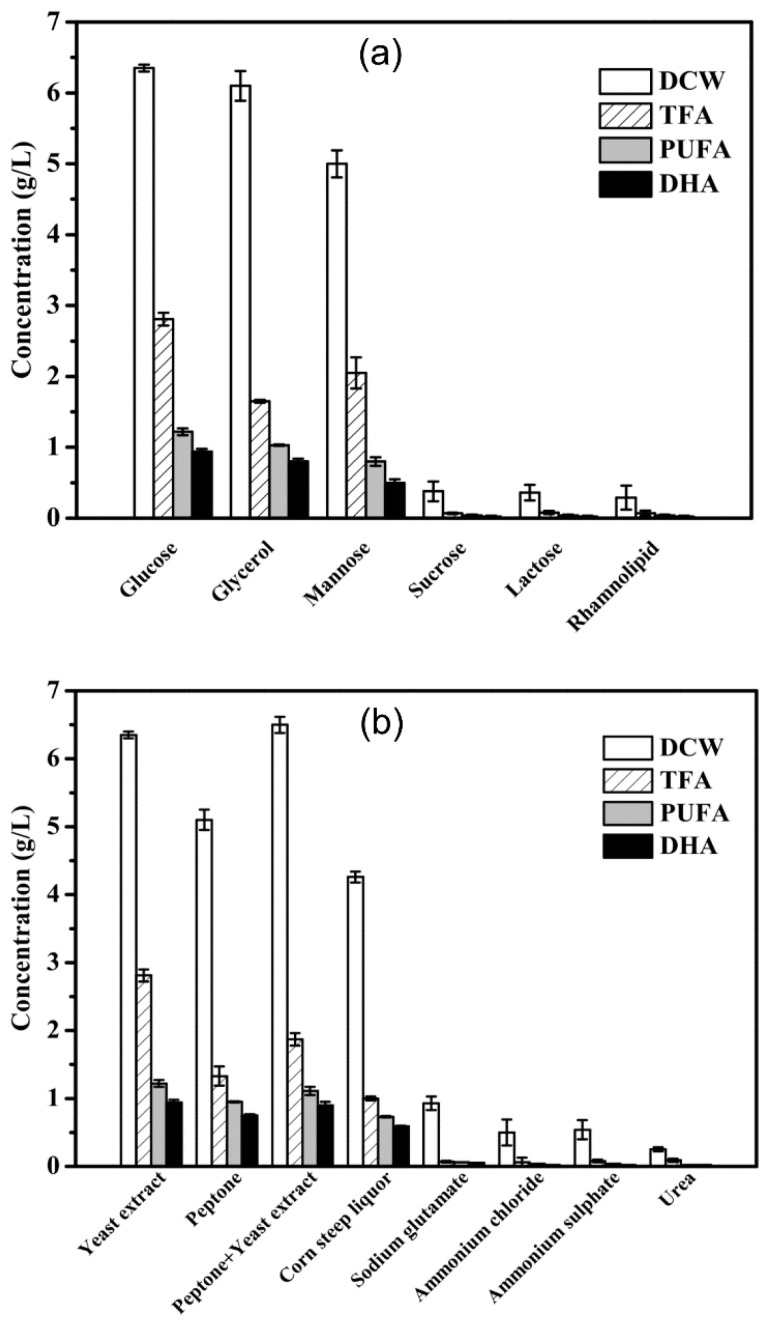
Biomass and fatty acids composition of mutant A78 cultivated on various (**a**) carbon sources (20 g/L) and (**b**) nitrogen sources (2.5 g/L). The bars represent the data for 96 h culture.

**Figure 4:**
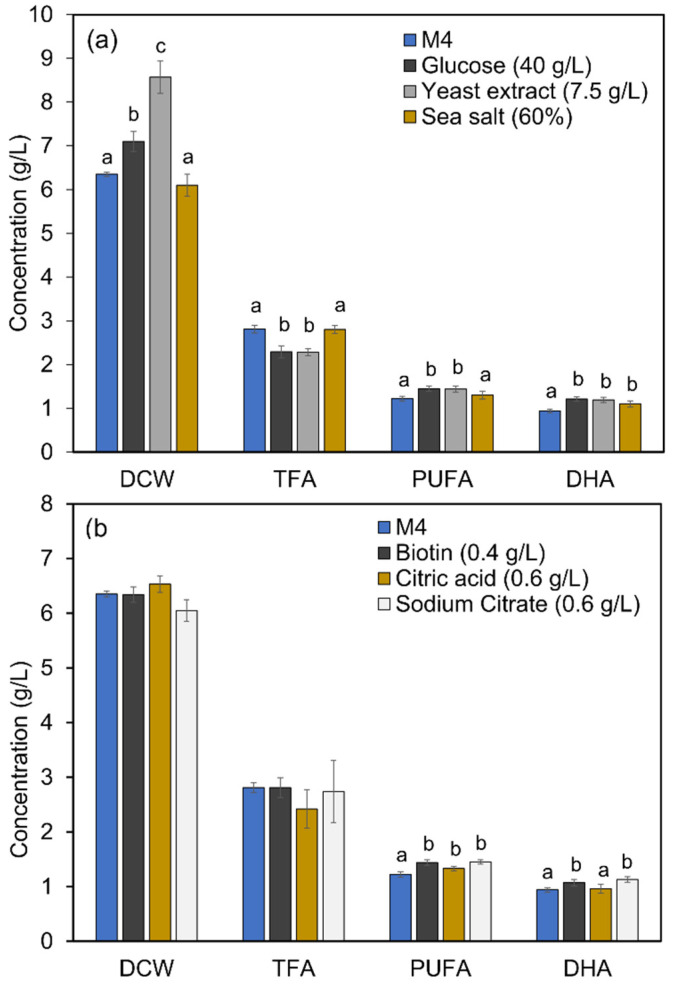
Biomass and fatty acid contents of mutant A78 cultivated in (**a**) M4 medium with optimal levels of glucose, yeast extract and sea salt and (**b**) M4 medium with optimal levels of biotin, citric acid and sodium citrate. Except for the studied factor in each OFAT experimental design, the other medium components and their concentrations were the same as that of the M4 medium. 60% seawater was equivalent to 19.8 g/L sea salt. The bars represent the data of 96 h culture. Significant (*p* < 0.01) differences are shown with different letters for each parameter.

The influence of various carbon and nitrogen sources on the DHA accumulation in mutant A78 was evaluated in this study. Glucose, glycerol and mannose were found as potential carbon sources (Figure 3a), while yeast extract, peptone, peptone plus yeast extract and corn steep liquor as potential nitrogen sources (Figure 3b), for achieving a significant amount of biomass and TFA. Glucose (40 g/L) and yeast extract (7.5 g/L) yielded the best DHA content, i.e., 1.21 and 1.19 g/L, respectively (Figure 4a). Further evaluation of various sea salt concentrations revealed that biomass and TFA contents of mutant A78 are not much affected by the change in the sea salt concentration, except for a slight increase in DHA production (Figure 4a).

**Figure 5:**
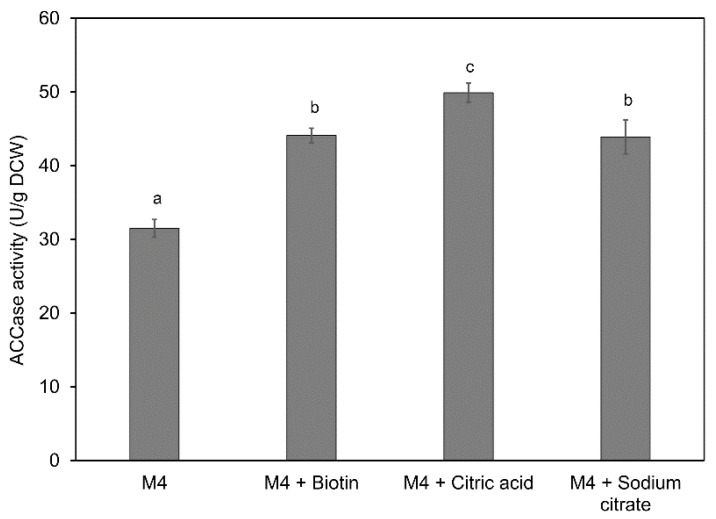
ACCase activity of mutant A78 cultivated in M4 medium supplemented with biotin, citric acid, or sodium citrate. The bars represent the data for 24 h culture. Significant (*p* < 0.05) differences are shown with different letters.

Furthermore, the addition of biotin, citric acid, or sodium citrate to the M4 medium showed no significant effect on the biomass and TFA contents of mutant A78 (Figure 4b). However, biotin and sodium citrate supplementation could increase the PUFA content to 1.44 and 1.45 g/L, respectively. The DHA contents achieved with biotin and sodium citrate supplementation were 1.07 and 1.13 g/L, an increase of 13.8 and 20.2%, respectively. Contrastingly, the addition of citric acid to the M4 medium had an insignificant effect on the DCW, TFA and DHA contents, except that it could increase the PUFA content by 9.0%. Further analysis of the ACCase activity of the mutant A78 revealed that biotin, citric acid and sodium citrate supplementation to M4 medium could increase the ACCase activity by 40, 58 and 38%, respectively (Figure 5).

### 2.5. Improved DHA Accumulation in Mutant A78 through Media Optimization

**Figure 6:**
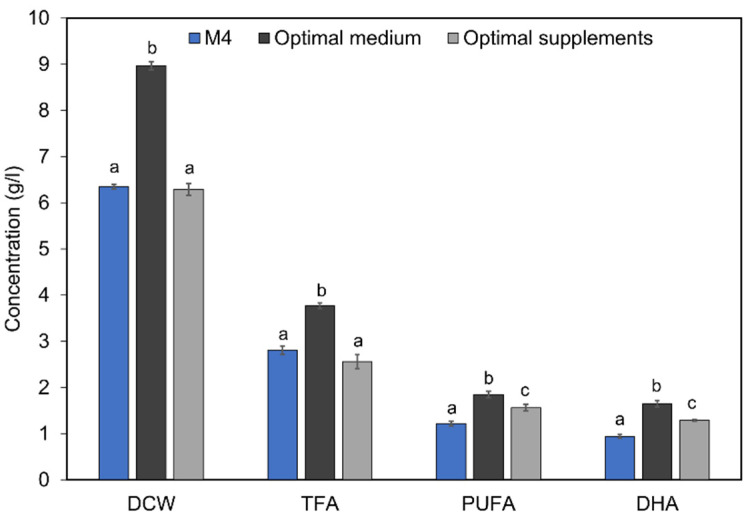
Comparison of biomass and fatty acids content of mutant A78 across different media conditions. The optimal medium/supplementation was determined from the orthogonal experiment. The optimal medium contained 40 g/L glucose, 7.5 g/L yeast extract and 50% of seawater, while the optimal supplements were 0.3 g/L biotin, 0.5 g/L citric acid and 0.5 g/L sodium citrate. The bars represent the data of 96 h culture. Significant (*p* < 0.01) differences are shown with different letters for each parameter.

To improve the DHA accumulation in mutant A78, two different sets of orthogonal experiments were conducted. The first set of experiments included three levels of glucose, yeast extract and sea salt (Table 2), while the second set of experiments included three levels of biotin (A), citric acid (B) and sodium citrate (C) (Table 3). The culture medium designed with the first set of orthogonal experiments, containing 40 g/L glucose (A2), 7.5 g/L yeast extract (B2) and 50% seawater (C1), yielded a DHA content of 1.65 g/L, which was 1.76-fold that with M4 medium (Figure 6). The initial C/N ratio of this optimal medium was 15.0. In addition, the DCW, TFA and PUFA contents also increased by 41.3, 34.2 and 51.6%, respectively (Figure 6). The contradiction was explained by the inhibition of high glucose concentration on cell growth and fatty acid synthesis. On the other hand, the culture medium designed with the second set of orthogonal experiments, containing 0.3 g/L biotin (A1), 0.5 g/L sodium citrate (B1) and 0.5 g/L citric acid (C1), could only increase the PUFA and DHA contents by 27.9 and 37.2%, respectively.

## 3. Discussion

ARTP has been widely used as a powerful and environmentally-friendly mutagenesis tool for generating mutant libraries and improving the yield of the target product from a variety of microorganisms [31,41]. Like other conventional mutagenesis methods, the treatment duration can be critical for achieving the desirable mutation efficiency [42]. In this study, *Schizochytrium* sp. PKU#Mn4 was found to be sensitive to the ARTP exposure time (Figure 1a). The optimal exposure time (40 s) of ARTP treatment to strain PKU#Mn4 was comparable with that of the microalgae such as *Crypthecodinium cohni* [39], *Spirulina platensis* [19], *Chlorella pyrenoidosa* [43] and *Spirulina platensis* [44]. However, compared with fungi, e.g., *Monascus purpureus* LQ-6 [45] and *Aspergillus niger* [46], the strain PKU#Mn4 was much more sensitive to the exposure time. These reported differences in the treatment duration could be attributed to the differential biochemical features of the microalgal and fungal cell walls that act as a natural barrier against environmental pressures [47]. *Schizochytrium* species have a thin non-cellulosic cell wall with galactose as the principal monosaccharide [48,49] and the thin cell wall is much easily penetrated by the high energy that leads to severe damage to DNA and proteins [50,51]. Our findings form the basis for future efforts to investigate how ARTP treatment affects the biochemical features of thraustochytrid species.

The modulatory or inhibitory role of chemicals in fatty acids biosynthesis has been commonly exploited to generate mutants with a great capacity for DHA or PUFA accumulation. For example, 2, 2’-Dipyridyl, an inducer of reactive oxygen species, was successfully used to screen mutants of *Schizochytrium* with a high antioxidant capacity [52]. Two inhibitors (isoniazid and triclosan) of enoyl-ACP reductase of Type II FAS pathway combined with cold stress were applied to screen mutants of *Aurantiochytrium* sp. Some other studies developed screening strategies based on malonic acid and iodoacetate acid to enrich the acetyl–CoA and NADPH pool, which resulted in mutants of *Schizochytrium* with 1.8-fold [53] and 2.0-fold change in DHA accumulation [54]. Unfortunately, the growths of most of these previously reported mutants of *Schizochytrium* were negatively affected [53,54,55]. In another study, a novel strategy of ARTP mutagenesis coupled with stepped malonic acid and zeocin resistance screening was applied to generate mutants of *Schizochytrium* sp. S31, which yielded a mutant mz-17 with a 1.8-fold in DHA [31]. Interestingly, in our study, the mutant A78, resulting from the clethodim-based screening, indicated that it cannot only accumulate 75.5% higher DHA but also had 41.3% higher biomass than that of the WT strain when cultivated in optimal medium (Figure 6). The improvement of both the biomass and lipid contents demonstrated the effectiveness of the clethodim-based screening compared with the aforementioned screening strategies. Our study provides a new screening strategy based on clethodim for efficient breeding of *Schizochytrium* with high DHA content but without much affecting the cell growth.

Earlier studies have demonstrated that ACCase activity plays a key role in fatty acids biosynthesis in lipogenic tissues of mammals [56] and cells of *Escherichia coli* [57]. ACCase activation via enzyme activators (e.g., citric acid and Mg^2+^) could enhance the microalgal lipid accumulation by almost 2-fold in *Chlorella vulgaris* [58]. The expression level of the ACCase gene in *Chlorella sorokiniana* was found to be upregulated by 2.1-fold from the exponential to stationary phase, indicating that ACCase gene expression is important in lipid biosynthesis [59]. Furthermore, transcriptomics analysis also indicated a closed association between upregulated ACCase and lipid accumulation in *Chlorella ellipsoidea* [60]. In this study, the two rounds of ARTP mutagenesis coupled with the ACCase inhibitor (clethodim)-based screening yielded the mutant A78. The increased DHA content of mutant A78 at 96 h of fermentation was consistent with its increased ACCase activity in the early exponential phase (24 h) of fermentation (Figure 4b and Figure 5), which suggested that DHA accumulation and ACCase activity are closely associated. Furthermore, when biotin was supplemented to the M4 medium, the ACCase activity of the mutant A78 was relatively increased, which indicated that biotin has a direct role in ACCase activity. As a cofactor for several essential carboxylase enzymes, biotin has been reported to be an important factor for the enzymatic activity of ACCase [61]. In addition, the enzyme ACCase is a biotin-dependent carboxylase and, therefore, biotin is important for its activity. In addition, biotin has been used as a media supplement to enhance fatty acids production in several thraustochytrid strains [62,63] and as a component of vitamin mixture [64]. Our findings suggest that ACCase would be an interesting target for engineering efficient strains of *Schizochytrium* in future studies.

Citric acid, a major intermediate of the tricarboxylic acid cycle, is known to play an important role in the biosynthesis of fatty acids [65]. However, in this study, citric acid did not much improve the DHA production (Figure 4b), probably because of the resulting acidic environment that has been reported unsuitable for cell growth and lipid accumulation of *Schizochytrium* sp. [66]. On the other hand, the aqueous solution of the sodium citrate is weakly alkaline and it can dissociate to form citrate ions, which can be catalyzed by the ATP:citrate lyase into acetyl-CoA—a key precursor used for lipid biosynthesis in oleaginous microorganisms [65]. Therefore, the addition of sodium citrate may not only buffer the culture environment but can also provide precursors for DHA synthesis. Overall, the results of citric acid and sodium citrate additions to the culture medium revealed that sodium citrate is a better supplement for improving the DHA accumulation of mutant A78.

## 4. Materials and Methods

### 4.1. Strain and Seed Culture

A previously isolated thraustochytrid strain, *Schizochytrium* sp. PKU#Mn4 [67], was used as the wild-type (WT) strain for ARTP mutagenesis. The strain was subcultured every two weeks on M4 solid medium containing (per liter) 20 g glucose, 1.5 g peptone, 1 g yeast extract, 33 g sea salt, 20 g agar and 1000 mL water. Seed culture was prepared in 100 mL glass flasks containing 40 mL M4 medium at 28 °C, 150 rpm for 24 h, which was used as the inoculum (5%, *v/v*) in all experiments. The C/N ratio of the initial M4 medium was 24.7, which was calculated based on the method described elsewhere [68].

### 4.2. Quantification of DCW, TFA and ACCase Activity

Cells were harvested by centrifuging at 4000 rpm for 10 min and then washed twice with sterile water. After lyophilization, the DCW of the harvested cells was estimated by the gravimetric method [67]. Lipid extraction, transesterification and quantification of fatty acids were performed following the procedures described in our previous studies [7,11]. The ACCase activity was detected by Acetyl-CoA Carboxylase ELISA Kit (Meimian Co., Jiangsu, China). All analyses were carried out in triplicate.

### 4.3. Sensitivity Assay of Schizochytrium sp. PKU#Mn4 to Clethodim

An 80 μL of 1000× 24 h grown PKU#Mn4 culture was spread on M4 medium plates, each containing a different concentration (0 to 100 μg/mL) of clethodim and cultivated at 28 °C for 72 h. For each concentration of clethodim, three replicate plates were set up. The individual colonies on the plates were counted manually and the lethality (%) was determined using Equation (1).

(1) $$Lethality\;\left(\%\right)=\;\frac{\mathrm{Control}\;\mathrm{colonies}\;-Survival\;colonies}{Control\;colonies}\times 100$$

### 4.4. ARTP Mutagenesis and Clethodim-Based Screening

A 10 μL seed culture suspension was evenly spread on the sterile metal plate and then treated with pure helium plasma under 120 W radiofrequency power input and 10 SLM helium flow rate using an ARTP Breeding Mutagenesis Machine (ARTP-M model, Yuanqing Tianmu Biotechnology Co., Ltd., Wuxi, China). The metal plate was immediately placed into a sterilized tube containing 1 mL of M4 medium after mutagenesis. An appropriate dilution of the resulting cell suspension was spread on M4 medium plates and cultivated at 28 °C for 72 h. The colonies were counted manually and the lethality of the ARTP treatment was calculated using Equation (1).

The mutants appearing on the plates were picked and inoculated into 100 mL flasks, each containing 40 mL of M4 medium and cultivated for 72 h. An 80 μL of that culture (1000×) was spread onto fresh M4 medium plates supplemented with clethodim (80 μg per mL of medium). The resulting plates were incubated at 28 °C for 72 h and the mutants appearing on the plates were picked and inoculated into 100 mL flasks, each containing 40 mL of M4 medium. The flasks were then incubated at 28 °C for 24 h and the resulting culture broth in each flask was used for the quantification of fatty acids. The colonies were first selected based on their colony size and growth on plates and then based on their DHA content in flask culture. The mutants with improved DHA content were selected as the starting strain for the second round of ARTP mutagenesis (40 s) and clethodim-based screening. The second round of mutagenesis and screening was conducted in the same manner as the first one, but with a slightly higher concentration (95 μg/mL) of clethodim for screening.

### 4.5. Genetic Stability of the Mutants

The mutant strains were first cultured on plates with M4 solid medium at 28 °C for 96 h. Then, several colonies were streaked onto new plates with M4 solid medium and subcultured for 96 h. Each selected mutant was subcultured six times, i.e., six generations. The mutant colonies from each subculturing were simultaneously inoculated in liquid M4 medium and incubated at 28 °C for 24 h. The resulting culture broth was used as the inoculum (5%, *v*/*v*) for the quantification of DCW and fatty acids. The genetic stability of the mutants was evaluated by monitoring the DCW and DHA yield across six generations.

### 4.6. Medium Optimization

The effects of medium components (carbon, nitrogen and sea salt) and supplements (biotin, citric acid and sodium citrate) on the biomass and DHA content of the mutant A78 were evaluated using a one-factor-at-a-time (OFAT) experimental design. Various carbon sources (glucose, glycerol, mannose, sucrose, lactose and rhamnolipid) at 20 g/L while nitrogen sources (peptone, yeast extract, peptone plus yeast extract, ammonium chloride, sodium glutamate, corn steep liquor, ammonium sulfate and urea) at 2.5 g/L were screened. To further obtain the optimal levels of the medium components and supplements, 9-run orthogonal experimental designs (Table 1 and Table 2) were formulated based on the significant factor levels determined from OFAT experiments. Triplicate samples were analyzed for each experiment. The orthogonal experiment was designed and statistically analyzed using ANOVA in SPSS Statistic 19 software. The C/N ratio of the initial optimal medium was calculated based on the method described elsewhere [68].

### 4.7. Statistical Analyses

The mean, SD and the test of significance (ANOVA) for each parameter were computed in SPSS Statistic 19.5. (IBM, New York, NY, USA)

## Figures and Tables

**Table 1:** Biomass, total fatty acids and DHA content of the wild-type strain PKU#Mn4 and its mutants obtained from the second round of ARTP mutagenesis and clethodim-based screening.

Strain/Mutant	Biomass (g/L)	TFA (g/L)	DHA (g/L)	DHA (g/g)	DHA/TFA (%)
WT	6.13	2.36	0.61	0.1	25.8
A10	6.42 (4.7%)	2.47 (4.7%)	0.87 ** (42.6%)	0.14 (40%)	35.2
A11	6.31 (2.9%)	2.40 (1.7%)	0.85 ** (39.3%)	0.13 (30%)	35.4
A17	6.47 (5.5%)	2.44 (3.4%)	0.87 ** (42.6%)	0.13 (30%)	35.7
A22	6.46 * (5.4%)	2.66 (12.7%)	0.92 ** (50.8%)	0.14 (40%)	34.6
A23	6.07 (−1.0%)	2.55 (8.1%)	0.87 ** (42.6%)	0.14 (40%)	34.1
A25	6.33 (3.3%)	2.60 (10.2%)	0.86 ** (41.0%)	0.14 (40%)	33.1
A29	6.71 * (9.5%)	2.66 (12.7%)	0.95 ** (55.7%)	0.14 (40%)	35.7
A36	6.49 * (5.9%)	2.51 (6.4%)	0.86 ** (41.0%)	0.13 (30%)	34.3
A49	6.59 * (7.5%)	2.40 (1.7%)	0.91 ** (49.2%)	0.14 (40%)	37.9
A54	6.28 * (2.4%)	2.51 (6.4%)	0.85 ** (39.3%)	0.14 (40%)	33.9
A74	6.75 ** (10.1%)	2.49 (5.5%)	0.95 ** (55.7%)	0.14 (40%)	38.2
A75	6.51 * (6.2%)	2.74 (16.1%)	0.87 ** (42.6%)	0.13 (30%)	31.8
A78	6.35 (3.6%)	2.81 * (19.1%)	0.94 ** (54.1%)	0.15 (50%)	33.5
A81	6.85 ** (11.7%)	2.48 (5.1%)	0.83 ** (36.1%)	0.12 (20%)	33.5
A89	6.28 (2.4%)	2.81 (19.1%)	0.95 ** (55.7%)	0.15 (50%)	33.8
A92	6.27 (2.3%)	2.72 (15.3%)	0.87 ** (42.6%)	0.14 (40%)	32.0
A99	6.70 ** (9.3%)	2.92 (23.7%)	0.98 ** (60.7%)	0.15 (50%)	33.6

Note: The data are provided for the samples collected at 96 h of cultivation. * *p* < 0.05; ** *p* < 0.01. The data in parenthesis indicate the percent increase compared with the WT strain.

**Table 2:** Factors and levels of the orthogonal experimental design for the optimal medium.

Experiment	A Glucose (g/L)	B Yeast Extract (g/L)	C Sea Salt (%) *
1	30	6.5	50
2	40	7.5	60
3	50	8.5	70

* 100% is equivalent to 33 g/L sea salt.

**Table 3:** Factors and levels of the orthogonal experimental design for the optimal supplementation.

Experiment	A Biotin (g/L)	B Citric Acid (g/L)	C Sodium Citrate (g/L)
1	0.3	0.5	0.5
2	0.4	0.6	0.6
3	0.5	0.7	0.7

## Data Availability

Not applicable.

## Supplementary Materials

The following are available online at https://www.mdpi.com/article/10.3390/md19100564/s1, Figure S1: Biomass and DHA contents of the wild-type strain PKU#Mn4 and its mutants resulting from the first round of ARTP mutagenesis and clethodim (80 μg/mL)-based screening. WT strain and mutants were cultured in an M4 medium and the culture samples were collected at 96 h for biomass and DHA quantification; Table S1: Palmitic acid content of the wild-type strain PKU#Mn4 and its mutants obtained from the second round of ARTP mutagenesis and clethodim-based screening.
