# Supplementary material for: ARTP Mutagenesis of Schizochytrium sp. PKU#Mn4 and Clethodim-Based Mutant Screening for Enhanced Docosahexaenoic Acid Accumulation
Source: Mar Drugs. 2021 Oct 7;19(10):564. doi: 10.3390/md19100564 (PMC8539320; doi:10.3390/md19100564)
Supplement: Supplementary file 1 [file marinedrugs-19-00564-s001.zip › marinedrugs-1402552-supplementary.pdf]

Supplementary Information

ARTP Mutagenesis of *Schizochytrium* sp. PKU#Mn4 and Clethodim-based Mutant Screening for Enhanced Docosahexaenoic Acid Accumulation

Lu Liu [1], Mohan Bai [1], Sai Zhang [1, 2], Jiantao Li [1], Xianhua Liu [1], Biswarup Sen [1, *],

Guangyi Wang [1, 3. *]

[1]Center for Marine Environmental Ecology, School of Environmental Science and Engineering, Tianjin University, Tianjin 300072, China

[2]Polar Research Institute of China, Shanghai 200136, China

[3]Key Laboratory of Systems Bioengineering (Ministry of Education), Tianjin University, Tianjin 300072, China

***Corresponding author.**

E-mail: bsen@tju.edu.cn; gywang@tju.edu.cn

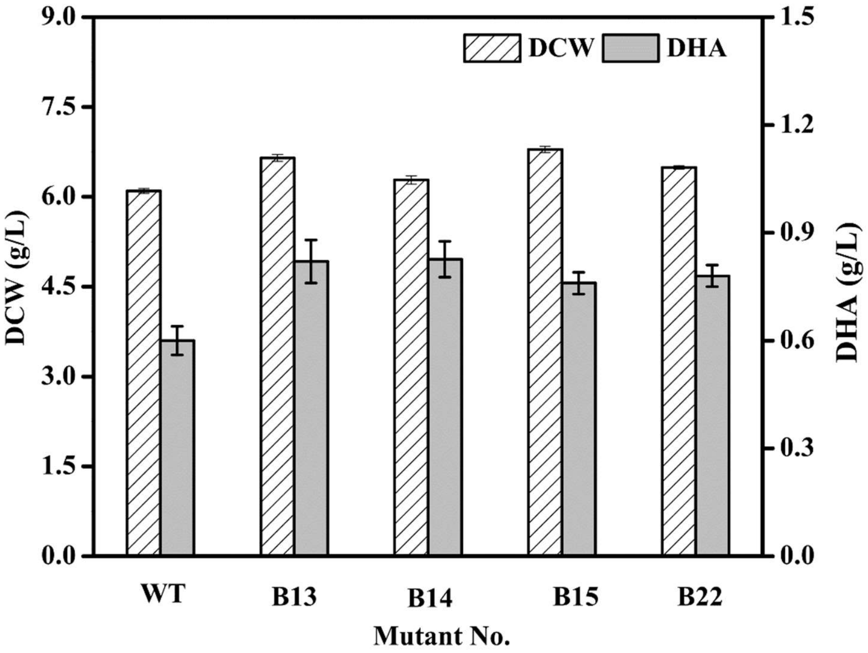

Figure S1. Biomass and DHA contents of the wild-type strain PKU#Mn4 and its mutants resulting from the first round of ARTP mutagenesis and clethodim (80 μg/mL)-based screening. WT strain and mutants were cultured in M4 medium and the culture samples were collected at 96 h for biomass and DHA quantification.

Table S1. Palmitic acid content of the wild-type strain PKU#Mn4 and its mutants obtained from the second round of ARTP mutagenesis and clethodim-based screening.

Mutant/Strain	C16:0
WT	0.88
A10	1.19
A11	1.20
A17	1.16
A22	1.30
A23	1.22
A25	1.29
A29	1.23
A36	1.18
A49	1.08
A54	1.21
A74	1.20
A75	0.87
A78	1.36
A81	1.24
A89	1.38
A92	1.40

Note: WT strain and mutants were cultured in M4 medium and the culture samples were collected at 96 h for fatty acids quantification.
